# Adolescents' Utilisation of Psychiatric Care, Neighbourhoods and Neighbourhood Socioeconomic Deprivation: A Multilevel Analysis

**DOI:** 10.1371/journal.pone.0081127

**Published:** 2013-11-15

**Authors:** Anna-Karin Ivert, Marie Torstensson Levander, Juan Merlo

**Affiliations:** 1 Faculty of Health and Society, Malmö University, Malmö, Sweden; 2 Unit of Social Epidemiology, Faculty of Medicine, Lund University, Malmö, Sweden; Wayne State University, United States of America

## Abstract

Mental health problems among adolescents have become a major public health issue, and it is therefore important to increase knowledge on the contextual determinants of adolescent mental health. One such determinant is the socioeconomic structure of the neighbourhood. The present study has two central objectives, (i) to examine if neighbourhood socioeconomic deprivation is associated to individual variations in utilisation of psychiatric care in a Swedish context, and (ii) to investigate if neighbourhood boundaries are a valid construct for identifying contexts that influence individual variations in psychiatric care utilization. Data were obtained from the Longitudinal Multilevel Analysis in Scania (LOMAS) database. The study population consists of all boys and girls aged 13–18 years (N=18,417), who were living in the city of Malmö, Sweden, in 2005. Multilevel logistic regression analysis was applied to estimate the probability of psychiatric care utilisation. The results from the study indicate that the neighbourhood of residence had little influence on psychiatric care utilisation. Although we initially found a variation between neighbourhoods, this general contextual effect was very small (i.e. 1.6 %). The initial conclusive association between the neighbourhood level of disadvantage and psychiatric care utilisation (specific contextual effect) disappeared following adjustment for individual and family level variables. Our results suggest the neighbourhoods in Malmö (at least measured in terms of SAMS-areas), do not provide accurate information for discriminating adolescents utilisation of psychiatric care. The SAMS-areas appears to be an inappropriate construct of the social environment that influences adolescent utilisation of psychiatric care. Therefore, public health interventions should be directed to the whole city rather than to specific neighbourhoods. However, since geographical, social or cultural contexts may be important for our understanding of adolescent mental health further research is needed to identify such contexts.

## Introduction

In everyday life, adolescents are exposed to a multitude of different social contexts and their mental health and development will be affected by these collective contexts in which they interact [1]. The most proximal context is the family, which is followed by contexts such as school, the peer-group and the neighbourhood. At a more distal level contexts such as culture and policy, will also influence adolescents’ health and behaviour. Over the past decade, a growing body of research has focused interest on the importance of the neighbourhood context for our understanding of health inequalities [2-4], and on the identification of the contextual boundaries that affect health and health-related behaviour [5-8]. When planning interventions to improve adolescent mental health or access to health care, it is important not only to identify the contextual characteristics that are associated with these outcomes. We also need to recognize the contexts in which individuals interact, and to disentangle the relative relevance of these different contexts for our understanding of individual differences in mental health and psychiatric care utilisation [8]. 

Mental health problems among children and adolescents constitute an important public health issue that affects many children and adolescents. Several studies have shown that the mental health of children and adolescents have worsen over the last decades, in Sweden [9,10] as well as in other countries [11,12]. About 20 percent of the world’s children and adolescents are estimated to suffer from mental health problems [13]. In Sweden about 30 percent of girls and 10 percent of boys aged 15 reported that they had felt depressed more than once a week during the last 6 months [14]. 

Mental health problems, and related mental health service use, among adolescents have been linked to a number of individual and/or family level factors such as adverse socioeconomic status at the individual level, ethnicity and family structure [15,16]. Moving beyond individual and family level attributes, several studies have identified an association between contextual factors at the neighbourhood level and both mental health problems and mental health service use in childhood and adolescence [17]. Concentrated socioeconomic disadvantage and different dimensions of community social capital are factors that have been identified as important for understanding differences in mental health among children and adolescents [18-21]. Socioeconomic deprivation and social capital have been hypothesised to affect mental health in children and adolescents through factors such as access to family advice and support, informal social networks with neighbours that might contribute to support, child-rearing methods, perceptions of risk and danger, and access to resources in the community (see for example 4,22,23). A study by van der Linden et al. [24] found that socioeconomic deprivation was also (positively) associated with mental health service use among children, and that this association was stronger in neighbourhoods with lower levels of social capital. The socioeconomic characteristics of the neighbourhood of residence may affect the utilisation of mental health care through the availability of healthcare options or through norms relating to which behaviours are viewed as normal and which behaviours warrant care provision [25]. 

However, in the analysis of neighbourhood effects on health, it is not sufficient to identify associations between neighbourhood characteristics and individual health. We also need to quantify the relevance of the neighbourhood construct as a whole for explaining individual level health differences. Basically, if the administrative boundaries used to defined neighbourhoods actually embrace a relevant social context that influences adolescent mental health, one would expect to find that a considerable proportion of adolescent differences in mental health were located at the neighbourhood level. In other words, we need to examine whether by knowing where an adolescent resides, we are able to differentiate who will develop mental health problems and who will not. For this purpose a methodological [26,27] and conceptual approach [7] has been developed. This approach has already been applied in several previous studies [28]. This approach shifts the focus from the specific contextual effects (i.e. the association between neighbourhood variables and the outcome) towards the general contextual effects. By studying general contextual effects we can show the extent to which the geographical constructs that we use for defining a neighbourhood determine individual outcomes (in this study psychiatric care utilization) without specifying any contextual characteristics other than the boundaries used for defining the neighbourhood [26]. This approach departs from traditional analyses by attempting to quantify heterogeneity around means and not only differences between averages. This problem has been referred to as the “Tyranny of the means” [29] or the “Mean centric approach” [30] and has been discussed in different scientific disciplines such as political science [30,31] and evolutionary biology [32]. Similar ideas have been developed in social epidemiology [7,28,33,34]. The key concept is that common measures of association are based on differences between mean values and that they are therefore abstractions that do not necessarily represent the heterogeneity of individual effects. 

Against this background, the present study has two central objectives, (i) to examine if neighbourhood socioeconomic deprivation is associated to individual variations in utilisation of psychiatric care in a Swedish context, and (ii) to investigate if neighbourhood boundaries are a valid construct for identifying contexts that influence individual variations in psychiatric care utilization. As far as we know, there is currently very little knowledge on this issue. We perform our study using a large database that includes all adolescents residing in the county of Skåne, Sweden, in 2005.

## Methods

### Study population

Our data are taken from the LOMAS (Longitudinal Multilevel Analysis in Scania) database that consists of unidentified information on all individuals living in Skåne, Sweden during the years 1968-2006. The database includes information on, amongst other things, all psychiatric care expenditures and use of psychotropic medication in out-patient and primary health care (dispensation at the pharmacy), as well as demographics, socioeconomic characteristics and country of birth. The data in LOMAS are collected from national registers (Statistics Sweden, The National Board of Health and Welfare), and from the county council in the Scania region (Region Skåne). The personal identification number that is assigned to each individual in Sweden was used by the Swedish authorities to link information from different registers. Once the linkage was done the database was anonymised. The current study population consists of all boys and girls aged 13–18 years (N=18,417), who were living in the city of Malmö, Sweden, in 2005. Malmö is Sweden’s third largest city with approximately 300,000 inhabitants. 

### Assessment of outcome variable

The outcome variable – utilisation of psychiatric care – is a combined measure consisting of (i) utilisation of psychiatric in- and outpatient care (measured as expenditures on psychiatric care in 2005) and (ii) dispensation of psychotropic medication (defined as all drugs with Anatomic Therapeutic Chemical (ATC) classification system codes starting with N05 or N06 [35]). This combined measure was created in order to capture not only the adolescents who received treatment for their mental health problems through the child and adolescent psychiatric care system, but also those who received such treatment via other sources, such as primary care.

### Assessment of individual and family variables (level 1)

Neighbourhoods may differ in terms of their composition with respect to factors associated with mental health and with treatment for mental health problems, and contextual variations may be an effect of these differences. Therefore, socioeconomic status (family income and level of education), family structure and parental country of birth were included to control for possible compositional effects. 

*Family income* was based on each adolescent’s weighted family income (i.e. household income divided by the number of family members, taking the ages of family members into consideration) at the end of 2004, and was analysed in tertiles, ranging from low-income to high-income families. We used the high-income group as the reference category. 

*Highest level of education in the family* was indicated by the parent’s educational attainment. Educational level was categorised into three groups based on years of schooling; nine years or less, 10-11 years, and 12 years or more of education. The twelve years or more category was used as the reference group. 

*Family structure* was categorised in three groups based on whether the adolescents were living with both birth/adoptive parents, with just one parent, or with neither of their parents. Adolescents living with both of their birth/adoptive parents were specified as the reference group. 

*Parental country of birth* was categorized according to the World Bank Classification of Country Economies [36], and distinguishes between high-income countries, upper-middle income countries, lower-middle income countries, and low-income countries. We separated Sweden from the high-income group and used Sweden as the reference category. If the parents were born in different countries, the mother’s country of birth was used for the classification (with the exception of cases where the father was born in Sweden, where the child was included in the reference category). 

The adolescents’ gender (with boys as the reference category) and age were also included in the analysis.

### Assessment of contextual characteristics (level 2)

In the present study, neighbourhoods were defined as small-area market statistics (SAMS) areas. SAMS refers to small administrative area units with an average population of 1000 residents. The boundaries of the units are drawn so as to include similar types of housing; this implies that SAMS neighbourhoods are comparatively homogeneous regarding socioeconomic structure [37]. A total of 315 neighbourhoods were originally included in the database, although we have only included neighbourhoods containing more than 20 adolescents in our analysis. The excluded neighbourhoods were for the most part industrial areas or sparsely populated neighbourhoods dominated by parks and recreational areas. The excluded adolescents did not differ from those included with regard to either the utilisation of psychiatric care or individual characteristics. 

The final sample consist of 17,729 adolescents (about 96 percent of the original sample) residing in 235 neighbourhoods (the number of adolescents per neighbourhood ranges between 20 and 703, median 55). This disparity in the number of adolescents living in different neighbourhoods is dealt with by the multilevel regression analysis [38] 

In order to characterise neighbourhood of residence, we created a measure of socioeconomic deprivation based on the total population of Malmö (N=271,264). The deprivation measure consists of aggregated data on the proportion of people with a weighted family income that was less than the city median (i.e. 114,352 SEK), the proportion of people receiving social welfare benefits, the proportion who were unemployed and the proportion of people with less than twelve years of education in each neighbourhood. We employed factor analysis to summarise these variables in a single construct. Using Principal Axis factoring, all four variables loaded on a single factor (loadings >0.45), which explained 65 percent of the total variance. Regression factor scores were calculated for the socioeconomic deprivation measure, yielding a continuous variable with a mean value of zero. A high value indicates a high level of socioeconomic deprivation. The deprivation measure was then divided into three groups by tertiles, which are referred to as a high-, mid-range and low level of deprivation. The low deprivation group was used as the reference category in the comparisons. 

### Ethics Statement

The construction of the record linkage database used in our study was approved by The Regional Ethical Review Board in Southern Sweden, The National Board of Health and Welfare and Statistics Sweden. Lund University signed a contract of confidentially with the Swedish Authorities. Active informed consent was waived as a requirement for the construction of the database.

### Statistical methods

Due to the hierarchical structure of the data, with the adolescents (first level) nested within neighbourhoods (second level) we applied multilevel logistic regression analysis to estimate the probability of mental health care utilisation [38,39].

The first step of the analysis involved fitting an empty model (i.e. a random intercept model). In this model, the probability of psychiatric care utilisation was purely a function of the adolescents’ residential neighbourhood, with the model showing the variance between neighbourhoods. The second model included individual variables based on household socio-demographic characteristics, along with age and gender, in order to control for a compositional effect and to estimate the role played by individual socio-demographic characteristics on the utilisation of psychiatric care. In our third model, we included the contextual level variable instead of the socio-demographic variables. This model indicates whether neighbourhood deprivation has any effect on the utilisation of psychiatric care. In the fourth and final model, individual, household socio-demographic and contextual variables were all included. 

In the interpretation of the multilevel analysis we distinguished between on the one hand individual-level and specific contextual effects and on the other general contextual effects [7]. Individual-level effects and specific contextual effects provide information about associations between individual/socio-demographic or contextual variables and the utilisation of psychiatric care, whereas the general contextual effects provide information about the degree to which the neighbourhoods studied explain individual differences in the utilisation of psychiatric care. 

To estimate individual and specific contextual effects, we calculated odds ratios (OR) and their 95 percent confidence intervals (95% CI). To measure the general contextual effects we calculated the variance partition coefficient (VPC), which in our case corresponds to the intra-class correlation (ICC). The ICC was calculated, using the latent variable method [38], as: ICC = neighbourhood variance/(neighbourhood variance+π^2^/3) *100. The ICC provides information on the proportion of the total individual variance in the probability of mental health care utilisation that can be found at the neighbourhood level. A small ICC (values close to zero) indicates that the neighbourhood of residence does not affect the utilisation of mental health care. 

Analyses were carried out using MLwiN 2.2, Centre for Multilevel Modelling, University of Bristol [40]. 

## Results

### Characteristics of the population

The characteristics of the study population are presented in Table 1. Approximately 6.5 percent of the adolescents received some type of treatment for mental health problems (psychiatric care and/or use of psychotropic medication) in the course of 2005. A total of 5.5 percent of the study population as a whole had utilised psychiatric care in the course of 2005 (the proportions utilising out- and inpatient care were 5.5 % and 0.6 % respectively). The corresponding figure for the dispensation of psychotropic medication was 2.6 percent. Mid-range deprived neighbourhoods had the highest proportion of adolescents who utilised some form of psychiatric care in the course of 2005 (7.6 %). 

**Table 1:** Characteristics of the population by tertiles of level of deprivation.

	Level of deprivation			
	Low	Mid-range	High	Total
	n=5926	n=5906	n=5897	N=17729
*Utilization of psychiatric care*	6.4	7.6	5.4	6.5
*Gender*				
Boy	51.3	50.1	51.6	51
Girl	48.7	49.9	48.4	49
*Age (mean)*	15.5	15.5	15.4	15.5
*Family income 2004*				
High	53.9	25.5	5.6	28.,4
Mid-range	29.3	36	18.5	27.9
Low	16.1	36.7	73.9	42.2
*Highest level of education in the family*				
≥12 years	77.3	60.2	47.6	61.7
10–11 years	19.1	28	23.6	23.6
≤ 9 years	3.3	11	26.6	13.6
*Family structure*				
Living with both parents	70.3	49.5	51.8	57.2
Living with only one parent	28.6	48.1	45.2	40.6
Not living with parents	1	2.4	3	2.1
*Parental country of birth*				
Sweden	86.8	64.9	22.2	58.1
High income country	1.7	3.2	3	2.6
Upper-middle income country	3.8	10.3	21.3	11.8
Lower-middle income country	4.8	16.8	40	20.5
Low income country	1	3	10.5	4.8

Values are percentages, unless otherwise indicated.

Compared to neighborhoods with a higher level of deprivation, those with a lower level of deprivation had greater proportions of adolescents with high family income and family education, adolescents living with both their parents, and adolescents with Swedish-born parents.

### Individual and specific contextual effects

Table 2 presents the associations between individual/family and contextual variables and psychiatric care utilisation. The individual/family-level model (Model II) indicates an increased probability of the utilisation of psychiatric care among girls (OR=1.50 CI: 1.33-1.69), and among adolescents from families with mid-range incomes (OR=1.22, CI: 1.04-1.43) and with an educational level of 10–12 years (OR=1.6, CI: 1.00-1.34). Not living with both biological/adoptive parents was associated with an increase in psychiatric care utilisation (OR 1.73, CI:1.51-1.98/OR=2.76 CI:1.83-4.16). Adolescents whose parents came from upper-middle, lower-middle and low income countries showed a lower probability of being treated for mental health problems compared with adolescents with Swedish-born parents (OR=0.56/0.60/0.38, CI:0.46-0.70/0.50-0.73/0.24-0.58). In Model III, neighbourhood level variables were included. This model shows that the probability of psychiatric care utilisation was 22 percent higher (CI: 1.04-1.43) among adolescents who lived in neighbourhoods with a mid-range level of deprivation. Model IV includes both individual/family and neighbourhood variables, and the association between the neighbourhood level of deprivation and the utilisation of psychiatric care is reduced to insignificance. The individual/family associations have only changed slightly in this final model.

**Table 2:** Multilevel logistic regression analysis showing specific associations between individual level and contextual level variables and utilization of psychiatric.

	Model I Empty model	Model II	Model III	Model IV
		OR (95% CI)	OR (95% CI)	OR (95% CI)
**Specific individual effects**				
*Gender*				
Boy		Reference		Reference
Girl		**1.50 (1.33-1.69)**		**1.51 (1.33-1.71)**
*Age*		0.97 (0.94-1.00)		0.98 (0.94-1.01)
*Family income 2004*				
High		Reference		Reference
Mid-range		**1.22 (1.04-1.43)**		**1.20 (1.01-1.41)**
Low		1.04 (0.88-1.24)		1.03 (0.86-1.25)
*Highest level of education in the family*				
≥ 12 years		Reference		Reference
10–11 years		1.16 (1.00-1.34)		1.15 (0.99-1.33)
≤9 years		1.21 (1.00-1.46)		1.20 (0.98-1.46)
*Family structure*				
Living with both parents		Reference		Reference
Living with only one parent		**1.75 (1.53-2.00)**		**1.73 (1.51-1.98)**
Not living with parents		**2.76 (1.83-4.16)**		**2.64 (1.73-4.02)**
*Parental country of birth*				
Sweden		Reference		Reference
High income country		0.79 (0.53-1-19)		0.80 (0.54-1.19)
Upper-middle income country		**0.56 (0.45-0.70)**		**0.57 (0.45-0.73)**
Lower-middle income country		**0.60 (0.50-0.73)**		**0.61 (0.50-0.75)**
Low income country		**0.38 (0.24-0.58)**		**0.38 (0.24-0.60)**
**Specific contextual effects**				
*Neighbourhood deprivation*				
Low			Reference	Reference
Mid-range			**1.22 (1.04-1.43)**	1.14 (0.97-1.34)
High			0.84 (0.71-1.00)	1.02 (0.82-1.28)

Values are odds ratios (OR) and 95% confidence intervals (CI).

### General contextual effects

Table 3 presents the general contextual effects. The first, empty model (Model I) shows that the neighbourhood of residence played a small but significant role in understanding psychiatric care utilisation among adolescents (ICC=1.6 %). Following adjustment for individual and family variables, in Model II the between-neighbourhood variance was reduced by 90 percent, indicating that a substantial amount of the variance between neighbourhoods was due to compositional factors, i.e. the characteristics of the adolescents living in the area. Adjusting for neighbourhood deprivation in Model III reduced the between-neighbourhood variance by 37 percent (by comparison with the empty model), indicating that some of the between-neighbourhood variation could be explained by the level of deprivation. In the fourth and final model, individual/family and contextual variables together reduced the between-neighbourhood variance by 96 percent (ICC in Model IV =0.1%).

**Table 3:** Multilevel analysis of variance showing general contextual effects on utilization of psychiatric care.

	Model IEmpty model	Model II	Model III	Model IV
Variance between neighbourhoods (SE)	0.052(SE 0.026)	0.005 (SE 0.005)	0.033 (SE 0.016)	0.002 (SE 0.001)
Intra-class correlation (ICC)	1.6%	0.2%	1.0%	0.1%
Explained variance (%)	-	90%	37%	96%

Analyses were also carried out for the utilisation of psychiatric care and the dispensation of psychotropic medication separately. Analysing utilisation and dispensation of psychotropic medication separately showed that there was a small neighbourhood effect on the utilisation of psychiatric care but not on the dispensation of psychotropic medication (data not shown).

## Discussion

The aim of this study was (i) to examine if neighbourhood socioeconomic deprivation is associated to individual variations in utilisation of psychiatric care in a Swedish context, and (ii) to investigate if neighbourhood boundaries are a valid construct for identifying contexts that influence individual variations in psychiatric care utilization. The results from the present study showed that the initial conclusive association between the neighbourhood level of socioeconomic deprivation and psychiatric care utilisation (specific contextual effect) disappeared following adjustment for individual and family level variables. Further, the results from the study indicate that the neighbourhood of residence had little influence on psychiatric care utilisation. Although we initially found a significant variation between neighbourhoods, this general contextual effect was very small (i.e. 1.6 %). 

Adolescent mental health is an important public health issue in Sweden, as it is in many other countries, and it is important to investigate the contextual determinants of adolescent mental health. When planning interventions to improve adolescent mental health or access to health care, it is important not only to identify contextual characteristics that are associated with these outcomes. It is equally important to identify the contexts where individuals interact, and to disentangle the relative relevance of these different contexts for understanding individual differences in mental health and the utilisation of psychiatric care. 

If the administrative boundaries used to define neighbourhoods actually constitute a relevant social context that influences adolescent mental health, a considerable proportion of adolescent differences in mental health would be found at the neighbourhood level. In previous research, the neighbourhood context has been identified as being important for our understanding of mental health problems and mental health care utilisation among children and adolescents [17,19,20,24]. The findings from the present study, however, partly contrast with the results from previous studies. The main reason for this discrepancy is that we evaluate the relevance of the context by measuring general contextual effects (i.e., ICC) rather than specific contextual effects only (i.e, OR) as most previous studies have done. This approach is analogous to the concept of discriminative accuracy used in other fields of Epidemiology [41-43]. Even though we initially found significant variation between neighbourhoods, this general contextual effect was very low (i.e. an ICC of 1.6 %), and the initial conclusive association between the neighbourhood level of disadvantage and psychiatric care utilisation (specific contextual effect) disappeared following adjustment for individual and family level variables.

Adolescents are simultaneously exposed to several contexts (e.g. neighbourhood, school, leisure-time and cultural associations, etc.). This means that we might consider contextual influences as a cross-classified multiple membership structure. Within the framework of this structure, the neighbourhood is only one of the many contexts that may condition mental health. The small general contextual effect identified in this study indicates that the neighbourhood context, at least when it is operationalized in terms of SAMS-areas, does not provide accurate information for discriminating adolescents utilisation of psychiatric care [42,43]. However, other geographical, social or cultural contexts might be more relevant for public health interventions aimed at reducing mental health problems among adolescents. The school context has been proposed as being important for the understanding of individual differences in health [44]. In this study, we unfortunately did not have access to information about the adolescents’ schools. However, a recent Swedish study of school effects on mental health (operationalized as self-inflicted injuries) found that less than one percent of the variation was due to the schools the individuals attended [45]. Another context that might be of importance for our understanding of adolescent differences in mental health is the geocultural context of the country of birth. Although ethnicity or parental country of birth have been identified as important factors in the understanding of variations in adolescents’ mental health and health care utilisation at the individual level, few studies have investigated the general contextual effects of country of birth [46,47]. In addition, a new methodological approach focused on the discriminatory accuracy [41] of different contexts might contribute to our interpretation of general contextual effects and how to disentangle the relative importance of different geographical, social or cultural contexts [42,43]. 

At the same time, based on the results from the present study, we cannot rule out the existence of either specific or general neighbourhood effects on adolescent mental health. It is possible that there is variation in the level of adolescents’ mental health problems between neighbourhoods that is not shown in register data. Several studies have shown that many children and adolescents with mental health problems do not receive treatment from the mental health services, and may as a consequence experience an unmet health care need [16,48]. This is an important area of research that needs to be elaborated on in future studies based on self- or parental-report data on mental health problems. However, the use of register-based data is also one of this study’s strengths. The register-based data made it possible to analyse data on psychiatric care utilisation relating to all adolescents living in the Scania region. In addition, the use of register-based data prevents biases that might result from self-reporting, i.e. some adolescents and/or their parents might not report symptoms despite service use. 

Furthermore, the results presented here may also be context specific to the Swedish society. Even though segregation has increased in Sweden over recent decades [49], Sweden is still considered to be a highly egalitarian country with relatively low levels of segregation [50] and a health care system that aims to allocate resources on equal terms to the whole population regardless of background or place of residence [51,52] This "buffering" effect of the Swedish health care system may be part of the explanation for the minor variation between neighbourhoods and the modest effect of socioeconomic deprivation on adolescent mental health care utilisation found in this study. However, in order to confirm this interpretation, our results need to be replicated in other samples of Swedish adolescents.

It should also be noted that in contrast to previous studies of neighbourhood effects on children’s and adolescents’ mental health and mental health service use, which have mainly focused on younger children [18,20,24], our sample is aged 13–18 years. It is possible that childhood mental health problems are more sensitive to the social context constituted by the neighbourhood of residence, since it is likely that younger children spend more time in their residential neighbourhood than adolescents. Among younger children, mental health problems and the utilisation of psychiatric care are more frequent among boys [53,54] and this type of mental health problems (i.e. externalising problems) might be more influenced by the social structure of the neighbourhood (e.g. through a lack of informal social control). At the same time however, running our multilevel analysis on children aged 7–12 resulted in similar specific and general contextual effects (data not shown). 

In addition, one of the limitations of the current study is its cross-sectional character and the consequent lack of information on longitudinal exposure to socioeconomic deprivation at the neighbourhood level. A recent Swedish study [55] found that the neighbourhood of residence during adolescence plays an important role in predicting future hospital admissions for drug abuse. Neighbourhood effects are not only dependent on the characteristics of the neighbourhood where the adolescents live today, but also on where they grew up and where their parents lived [56,57]. 

## Conclusions

Our results suggest the neighbourhoods in Malmö (at least measured in terms of SAMS-areas), does not provide accurate information for discriminating adolescents utilisation of psychiatric care. The SAMS-areas appears to be an inappropriate construct of the social environment that influences adolescent utilisation of psychiatric care. Therefore, public health interventions should be directed to the whole city rather than to specific neighbourhoods. However, since geographical, social or cultural contexts may be important for our understanding of adolescent mental health further research is needed to identify such contexts.
